# Identifying no-harm incidents in home healthcare: a cohort study using trigger tool methodology

**DOI:** 10.1186/s12913-020-05139-z

**Published:** 2020-04-06

**Authors:** Marléne Lindblad, Maria Unbeck, Lena Nilsson, Kristina Schildmeijer, Mirjam Ekstedt

**Affiliations:** 1 School of Engineering Sciences in Chemistry, Biotechnology and Health, Royal Institute of Technology, Stockholm, Sweden; 2 Department of Healthcare Sciences, Ersta Sköndal Bräcke University College, Stockholm, Sweden; 3 Department of Neurobiology, Care Sciences and Society, Karolinska Institutet, Stockholm, Sweden; 4 Acute and Reparative Medicine Theme, Karolinska University Hospital, Stockholm, Sweden; 5 Department of Anesthesiology and Intensive Care, Department of Biomedical and Clinical Sciences, Linköping University, Linköping, Sweden; 6 Department of Health and Caring Sciences, Faculty of Health and Life Sciences, Linnaeus University, Kalmar, Sweden; 7 Department of Learning, Informatics, Management and Ethics, Karolinska Institutet, Stockholm, Sweden

**Keywords:** No-harm incident, Trigger tool, Retrospective record review, Home healthcare, Patient safety

## Abstract

**Background:**

Patient safety in home healthcare is largely unexplored. No-harm incidents may give valuable information about risk areas and system failures as a source for proactive patient safety work. We hypothesized that it would be feasible to retrospectively identify no-harm incidents and thus aimed to explore the cumulative incidence, preventability, types, and potential contributing causes of no-harm incidents that affected adult patients admitted to home healthcare.

**Methods:**

A structured retrospective record review using a trigger tool designed for home healthcare. A random sample of 600 home healthcare records from ten different organizations across Sweden was reviewed.

**Results:**

In the study, 40,735 days were reviewed. In all, 313 no-harm incidents affected 177 (29.5%) patients; of these, 198 (63.2%) no-harm incidents, in 127 (21.2%) patients, were considered preventable. The most common no-harm incident types were *“fall without harm,” “deficiencies in medication management,”* and *“moderate pain.”* The type *“deficiencies in medication management”* was deemed to have a preventability rate twice as high as those of *“fall without harm”* and *“moderate pain.”* The most common potential contributing cause was *“deficiencies in nursing care and treatment, i.e., delayed, erroneous, omitted or incomplete treatment or care.”*

**Conclusion:**

This study suggests that it is feasible to identify no-harm incidents and potential contributing causes such as omission of care using record review with a trigger tool adapted to the context. No-harm incidents and potential contributing causes are valuable sources of knowledge for improving patient safety, as they highlight system failures and indicate risks before an adverse event reach the patient.

## Introduction

As home healthcare is a growing and increasingly complex arena, there is an urgent need to expand patient safety research of home healthcare settings [1]. Evidence from past research, mainly deriving from in-hospital settings, are not easily transferred to home healthcare settings, as the preconditions for safety in the context of home healthcare, in essential aspects are fundamentally different from in-hospital care [2, 3]. Safety in home healthcare is challenged by unclear boundaries of responsibilities and communication paths between different care providers, patients, and family caregivers [4, 5]. Safety issues can also relate to a patient’s ability to process complex information and manage self-care on their own, [6, 7] as well as to the uncontrolled environment, e.g., a patient’s private home, which is usually not designed for provision of care [8].

Structured retrospective record review (RRR) using a trigger tool (TT) is a valid and thorough method for measuring the incidence of AEs, [9–11] and has successfully been developed and used for identifying adverse events (AEs) in different kind of specialties in in-hospital care [12–14]. One well described TT is the Global Trigger Tool (GTT) [15], commonly used in acute inpatient care. RRR using TT has the potential to give an overview of the incidence, nature, preventability and consequences of AEs, hence providing an opportunity to identify risk areas and learn from past failures. Although TT has most commonly been used in hospital settings, a few studies used similar methodology for detecting patient safety issues in home healthcare [16–18].

Recently the TT method was developed and validated for home healthcare in Swedish context [19]. A total of 600 home healthcare records were reviewed and AEs occurred in 37,7% of the records [20]. Home healthcare in Sweden is highly differentiated between different caregivers and professional roles and the documentation is thus separated in different systems and by different traditions (i.e. social care and healthcare) [21]. As poor documentation quality limits the detectability of AEs in retrospective record review, [22] there is reasons to believe that there are ‘blind spots’ where it is difficult to identify risk areas by focusing on AEs solely.

Modern system safety approach to patient safety suggests that both successful results and failure derives from the same system [23–25]. Hence, drawing attention to no-harm incidents may provide important information on areas for improvement, and learning from how emerging risks are anticipated and mitigated before discernible harm is caused. Applying such approach to identification of potential risks, in parallel to learning from past AEs, is necessary in order to gain knowledge and understanding of how to improve patient safety in home healthcare [26].

However, identifying no-harm incidents are usually excluded in RRR methods [15] Only two previous studies have been identified using record review to identify no-harm incidents in hospital care [27, 28]. We thus hypothesize that it is feasible to identify no-harm incidents in parallel to AEs using TT methodology also in home healthcare. Further, that it in parallel with the identification of AEs will give complement and valuable information about brittleness in patient safety areas.

Thus, this study aimed to explore the cumulative incidence, preventability, types, and potential contributing causes of no-harm incidents that affected adult patients admitted to home healthcare, using a TT methodology.

## Methods

### Design

This was a cohort study using RRR and TT methodology, which encompassed 600 randomly selected healthcare records from ten home healthcare organizations across Sweden, included by convenience sampling. The identification of both AEs and no-harm incidents in the home healthcare context was conducted at the same time when reviewing the records. Sample size calculation was based on the identification of AEs [19]. The method and the identification of AEs is described in detail elsewhere [19, 20].

### Setting and sample

The Swedish Health and Medical Services Act, [29] designed to ensure that everyone living in Sweden has access to good healthcare, gives the county councils/regions and municipalities considerable freedom in organizing care. The organization of home healthcare services differs within the country and the provision of home healthcare in an area can be the responsibility of either the county council or the municipality. Physicians are always employed by the county councils, with which the municipalities collaborate. There are both public and private home healthcare facilities, but they are generally publicly funded. Home healthcare usually means care provided by licensed healthcare professionals and does not include home care organizations with unlicensed staff administering social care.

In this study, seven of the review teams were employed by municipalities and three worked in home healthcare for county councils. Each review team consisted of one to three registered nurses and one or two physicians, in total 28 clinicians. The ten review teams represented nine different regions across Sweden.

### Definitions

In the RRR process, a no-harm incident was an event caused by healthcare or social care that reached the patient and could have led to an AE, but resulted in no discernible harm [30]. A preventable no-harm incident was defined as an event that could have been prevented if adequate actions had been taken during the patient’s contact with healthcare or social care. No-harm incidents related to acts of either omission or commission were included.

### Inclusion and exclusion criteria

All patients aged 18 years or older admitted to home healthcare at the studied units during 2015 were included in the sample from where the randomly selected records were retrieved. The record review covered a maximum of 90 days from the date of a patient’s admission to home healthcare (index admission). If a patient had been discharged from home healthcare and readmitted within the 90-day period, the review of the record continued.

To be included as a no-harm incident, the following criteria had to be met; the no-harm incident occurred and was detected in the home healthcare records during the index admission, i.e., within 90 days after enrolment in home healthcare, regardless of caregiver.

Randomization was performed by one of the researchers (MU), using an online randomizer, to ensure it was carried out in the same way for all review teams. Oversampling was performed by 10 records per team. If a patient in the random sample was receiving limited home healthcare, e.g., only blood pressure measurement, this patient's admission was replaced by another randomly selected admission.

### Review process

Before review start, each review team member underwent a mandatory one-day education in the TT methodology, including practical training in reviewing healthcare records and discussion of detailed examples. A trigger manual and a study manual were used as the basis for decision-making during the review process.

Each review team reviewed 60 randomly selected home healthcare records from their own organization. The RRR was conducted in two stages, a primary and a secondary review. Usually, the registered nurses in each team performed the primary and secondary reviews and then discussed their findings with the physicians until consensus was reached. In some teams, the physicians carried out some of the primary and/or secondary reviews. In the primary review, the reviewer documented demographic data and screened for the presence of the 38 predefined triggers in each healthcare record [19]. A trigger is a word or a sentence in a record that could indicate that a no-harm incident had occurred, such as *“fall”* or *“insufficient planning, coordination, communication, and/or information.”* For each identified trigger, in parallel to screening for AEs, [20] the reviewer assessed whether or not it reflected the presence of a potential no-harm incident. Ten percent of the records in the primary review process were evaluated concerning inter-rater reliability. The inter-rater reliability of the primary reviewers’ judgements concerning if a record was to be forwarded to secondary review was *κ* = 0.801 (substantial) [19]. If a potential no-harm incident was found, the record went forward to secondary review.

The reviewer sorted the different triggers into potential no-harm incidents, as several triggers can be related to a specific no-harm incident. Each event was then reviewed separately. Initially, the reviewer assessed whether the event was associated with healthcare/social care using a 4-point scores; 1) the event was not related to healthcare/social care; 2) the event was probably not related to healthcare/social care; 3) the event was probably related to healthcare/social care; 4) the event was related to healthcare/social care. A score of 3 or 4 on the Likert scale was required for the event to went forward in the review process. A score of 1 or 2 was for example an event that was assessed as a consequence of the disease’s development or considered to be socially acquired. A similar 4-point scale was used to assess if the event was considered preventable or not and was deemed from the patient perspective. For example, a “fall without harm” was considered to be preventable if no risk assessment, or risk identification was carried out, or if preventive measures of predictable risks in the physical care environment (i.e home environment) had not been discussed with the patient.

“Unplanned hospitalization” due to inability from home healthcare personnel to “handle medical-technical equipment” in the patient’s home was considered preventable through adequate education. For each trigger the study manual gave examples to guide decisions for preventability.

The National Coordinating Council for Medication Error Reporting and Prevention criteria (NCC MERP) were used to assess severity [31]. NCC MERP consists of nine categories (A–I). In the present study, category C (an event that reached the patient but did not cause harm) and category D (an event that reached the patient and required monitoring or intervention to ensure that no harm occurred) were included, i.e., were seen as no-harm incidents.

Data were also collected regarding interventions and potential contributing causes linked to the no-harm incidents. Several interventions and potential contributing causes per no-harm-incident could be selected from a predefined list. The contributing causes we used have been evolved over time. They are originally derived from HMPS studies and other classification systems as the Swedish clinical incident reporting system.

All no-harm incidents that were detected in the home healthcare records during the review were included, regardless of if they originated from home healthcare or other healthcare or social care organizations. The record systems were generally computerized, but documentation routines and access to the full records or parts thereof differed between the teams.

### Data analysis

The data were analyzed descriptively (presenting cumulative incidence, range and percent). The chi-squared (χ^2^) test was used to analyze differences between men and women and between patients aged 80 years or older and patients younger than 80 years. Differences between groups were considered to be statistically significant if the *p* values were < 0.05. The SPSS statistics program version 24 and Statistica 64 V.13 were used to perform calculations.

## Results

The study sample consisted of 600 home healthcare records and a total of 40,735 days was reviewed. The mean number of reviewed days per record was 67.9 (SD 30.9, median 90 days, range 1–90). The mean age of the patients was 78.2 years (SD 12.4, median 80.5 years, range 20–99), with 53.3% females (*n* = 320). The most common medical conditions were malignancy and cardiovascular diseases. Most patients had several diagnoses and medical needs. The most common reason for admission was a need of medication assistance (*n* = 233, 38.8%), followed by palliative care (*n* = 144, 24%). The distribution was equal between patients who lived single and patients who were cohabiting, 265 (44.2%) versus 257 (42.8%). For the remaining 28 patients (4.7%), living situation was not documented in the records.

**Fig. 1:**
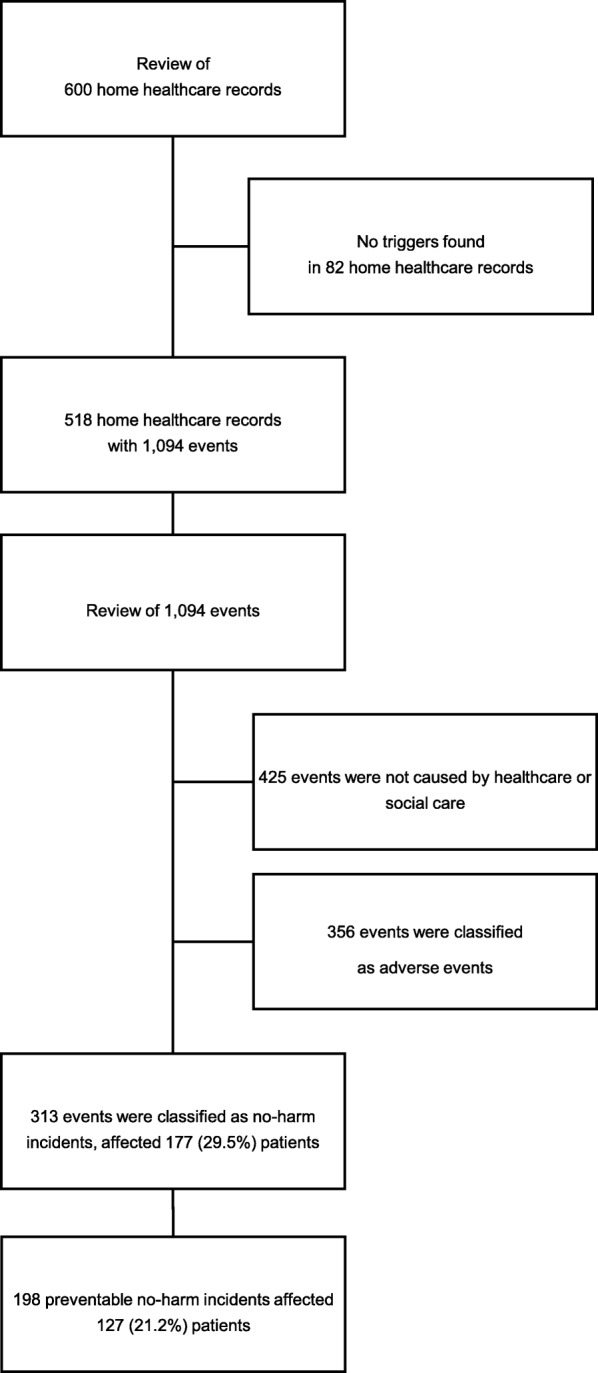
Flowchart of retrospective record review of 600 randomly selected home healthcare records

A flowchart of the 600 home healthcare records is provided in Fig. 1. A total of 1094 events were forwarded to secondary review. An event relates to either an AE or a no-harm incident. The reviewers judged 313 of these as no-harm incidents, affecting 177 (29.5%) patients with a median of three (range 1–16) no-harm incidents per affected patient. Of the total no-harm incidents, 198 (63.3%) were considered preventable, affecting 127 (21.2%) patients. The number of patients affected by more than one no-harm incident was 66 (11.0%), with 35 (53.0%) affected by more than one preventable no-harm incident. The number of no-harm incidents per 100 patients was 52, and the corresponding number for preventable no-harm incidents was 33. The number of no-harm incidents and preventable no-harm incidents per 1000 patient days was 7.7 and 4.9, respectively. There was no difference in the rate of no-harm incidents between men and women (*p* = 0.61), or between patients aged 80 years or older and younger patients (*p* = 0.84).

Using the NCC MERP scale, 186 (59.4%) no-harm incidents were judged as an event that reached the patient but did not cause harm (category C) and 127 (40.6%) were judged as an event that reached the patient and required monitoring or intervention to ensure that no harm occurred (category D). Of these 55.4% respectively 74.8% were considered preventable.

The types of no-harm incidents and their preventability are shown in Table 1. The most common type of no-harm incident was *“fall without harm,”* which was considered preventable to a lesser extent than most other types of no-harm incidents. The second most common type, *“deficiencies in medication management,”* resulted in patients not receiving the correct prescribed dose of their medication. *“Deficiencies in medication management”* were deemed to have a preventability rate twice as high as those of *“fall without harm”* and *“moderate pain.”* The incident type *“other”* encompasses a diverse set of events, such as moderate dehydration and a patient removing his/her gastric feeding tube.

**Table 1:** Types of no-harm incidents and their preventability

Types of no-harm incidents	No-harm incidents n (%)	Preventable no-harm incidents n (%)
Fall without harm	127 (40.6)	52 (40.9)
Deficiencies in medication management	62 (19.8)	61 (98.4)
Not administered in accordance with prescription	32 (51.6)	32 (100)
Prescription – missing or unclear	15 (24.2)	14 (93.3)
Distributed drug or dose – incorrect or missing	12 (19.4)	12 (100)
Deficient storage at home	3 (4.8)	3 (100)
Moderate pain	24 (7.7)	12 (50.0)
Moderate constipation	14 (4.5)	13 (92.8)
Deficiencies in communication and coordination	12 (3.8)	12 (100)
Moderate psychological impairment	12 (3.8)	10 (83.3)
Affected laboratory values	10 (3.2)	7 (70.0)
Moderately distended urinary bladder ^a^	9 (2.9)	3 (33.3)
Flaws in taking blood sample	6 (1.9)	6 (100)
Moderate diarrhea	5 (1.6)	1 (20.0)
Moderate medication side effect	5 (1.6)	2 (40.0)
Blockage in subcutaneous venous port	4 (1.3)	4 (100)
Moderate acute confusion	3 (1.0)	3 (100)
Moderate vomiting	3 (1.0)	0 (0)
Moderate deterioration in health status	3 (1.0)	1 (33.3)
Other	14 (4.5)	11 (78.6)
Total	313 (100)	198 (63.3)

^a^ An estimated volume of between 500 and less than 1000 ml of urine in the bladder on one occasion is considered a no-harm incident

There was no difference in outcome for the two most common types of incidents, *“fall without harm”* and *“deficiencies in medication management,”* between men and women (*p* = 0.14 and *p* = 1.0, respectively) or between patients aged 80 years or older and patients younger than 80 years (*p* = 0.19 and *p* = 0.54, respectively). *“Moderate pain”* was more common among men than women (*p* = 0.04) and also more common among patients younger than 80 years than those aged 80 years or older (p = 0.04).

The total documented number of interventions following the no-harm incidents was 377. That is higher than the number of no-harm incidents, as the reviewers could choose more than one intervention for each no-harm incident. The most common documented interventions were *“extra blood samples, procedures, nursing care and treatments”* (*n* = 129). Other documented interventions included *“delays in assessments, investigations and treatments”* (*n* = 35) and “*extra visits to outpatient care”* (*n* = 23). For a large proportion of the no-harm incidents, no inventions were documented (*n* = 120).

Of the total no-harm incidents, 259 (82.7%) derived from home healthcare and 51 (16.3%) from caregivers outside home healthcare: ten (3.2%) from outpatient care, 17 (5.4%) from in-hospital care and 24 (7.7%) from social care. No documentation was found on the origin of the three remaining no-harm incidents. The most common type of no-harm incident originating from caregivers outside home healthcare was related to *“deficiencies in medication management,”* (*n* = 18, 35.3%).

Different categories of potential contributing causes influencing the no-harm incidents are listed in Table 2. Each category consists of several subcategories with a more explicit character. A majority of the no-harm incidents had multiple contributing causes whereas 51 (16.3%) no-harm incidents had a single and for 25 (8.0%) no contributing causes were found by the review team. Many of the potential contributing causes were related to *“deficiencies in nursing care, treatment, and diagnostic”.*

**Table 2:** Potential contributing causes influencing no-harm incidents

Type of contributing causes	n (%)
**Deficiencies in nursing care, treatment, and diagnostics**	629 (47.9)
Nursing care – delayed, erroneous, omitted, incomplete	168 (26.7)
Treatment – delayed, erroneous, omitted, incomplete	143 (22.7)
Observation – delayed, erroneous, omitted, incomplete	100 (15.9)
Follow-up of care/treatment – delayed, erroneous, omitted, incomplete	91 (14.5)
Performance of task – deficient	58 (9.2)
Diagnostics/examination – delayed, erroneous, omitted, incomplete	43 (6.8)
Paramedical care – delayed, erroneous, omitted, incomplete	17 (2.7)
Acting outside own area of competence	4 (0.6)
Checking/labelling of samples, examination, patient identity – deficient	3 (0.5)
Preparation of patient ahead of operation, examination – inadequate	2 (0.3)
**Deficiencies in communication, information, and collaboration**	257 (19.5)
Communication/information – deficiencies between different care providers	54 (21.0)
Collaboration/continuity/care planning – deficiencies within the unit	48 (18.7)
Communication/information – deficiencies within own unit/care provider	44 (17.1)
Communication/information – deficiencies in relation to patient/next-of-kin	36 (14.0)
Collaboration/continuity/care planning – deficiencies between units	24 (9.3)
Information – deficiencies in acting on available information	22 (8.6)
Interpretation of information – deficiencies	10 (3.9)
Attention and/or having expected staff not visit – deficiencies, delays	9 (3.5)
Discharge planning – deficiencies	6 (2.3)
Communication/information – deficiencies regarding decision-making conversations	3 (1.2)
Language barriers	1 (0.4)
**Deficiencies in the organization**	204 (15.5)
Routines/guidelines – lacking, deficient, have not been observed	81 (39.7)
Routines/guidelines – unknown	24 (11.8)
Deficiencies in competence and experience	23 (11.3)
General organizational flaws	20 (9.8)
Deficient relational continuity	13 (6.4)
Resources – lacking	12 (5.9)
Distribution of responsibilities – unclear	12 (5.9)
Wrong level of care	9 (4.4)
Management – deficiencies	7 (3.4)
Availability – lacking	3 (1.5)
Physician with patient responsibility not appointed	1 (0.5)
**Deficiencies in medication management process**	168 (12.8)
Medication – prescription – delayed, erroneous, omitted, incomplete	62 (36.9)
Medication – distribution – delayed, erroneous, omitted, incomplete	58 (34.5)
Medication – preparation – delayed, erroneous, omitted, incomplete	29 (17.3)
Medication – lacking in-depth presentation of drug	14 (8.3)
Medication – side effects	5 (3.0)
**Technical device issues**	19 (1.4)
Medical equipment, tool – handling errors, lacking knowledge on use	12 (63.2)
Medical equipment, tool – insufficient access, defects, insufficient maintenance	6 (31.6)
IT-related – problem with IT system, insufficient access, handling errors	1 (5.3)
**Other**	37 (2.8)
Not apparent from record	33 (89.2)
Other cause	4 (10.8)
Total	1314 (100)

The number of potential contributing causes is higher than the number of no-harm incidents because the reviewers were allowed to choose more than one alternative for each no-harm incident

The most common potential contributing causes of the three most common no-harm incidents (listed in Table 3) were related to “*delayed, erroneous, omitted or incomplete observations*”, “*treatment, nursing care*” and “*deficient performance of tasks*”, which in all aspects emphasizes the organizational and the human factor.

**Table 3:** The three most common types of no-harm incidents and their most common potential contributing causes

Types of no-harm incidents	Potential contributing causes	Number
Fall without harm (*n* = 127, 40.6% of all no-harm incidents)	Nursing care – delayed, erroneous, omitted, incomplete	66
	Observation – delayed, erroneous, omitted, incomplete	48
	Follow-up of care/treatment – delayed, erroneous, omitted, incomplete	36
	Not apparent from record	29
	Routines/guidelines – lacking, deficient, have not been observed	25
	Collaboration/continuity – deficiencies within the unit	23
	Treatment – delayed, erroneous, omitted, incomplete	16
	Communication/information – deficiencies in relation to patient/relatives	16
	Communication/information – deficiencies between different care providers	16
Deficiencies in medication management (*n* = 66, 21.1% of all no-harm incidents)	Treatment – delayed, erroneous, omitted, incomplete	63
	Performance of task – deficient	46
	Medication – distribution – delayed, erroneous, omitted, incomplete	46
	Routines/guidelines – lacking, deficient, have not been observed	43
	Communication/information – deficiencies *between* different care providers	31
	Follow-up of care/treatment– delayed, erroneous, omitted, incomplete	25
Moderate pain (*n* = 24, 7.7% of all no-harm incidents)	Treatment – delayed, erroneous, omitted, incomplete	15
	Nursing care – delayed, erroneous, omitted, incomplete	10
	Diagnostics/examination – delayed, erroneous, omitted, incomplete	7
	Medication – prescription – delayed, erroneous, omitted, incomplete	7
	Follow-up of care/treatment– delayed, erroneous, omitted, incomplete	7
	Observation – delayed, erroneous, omitted, incomplete	7

The number of potential contributing causes is higher than the number of no-harm incidents because the reviewers were allowed to choose more than one alternative for each no-harm incident

## Discussion

Our study confirms that the TT methodology can be useful for patient safety work to detecting risk areas before they cause harm to the patients. The identification of no-harm incidents and contributing causes, conducting in parallel with AEs, [19] will give complement and valuable information about brittleness in patient safety areas.

The adapted TT for home healthcare revealed that no-harm incidents affected almost every third patient and just over half of the incidents were considered preventable. The most common types of no-harm incidents were *“fall without harm,” “deficiencies in medication management,”* and *“moderate pain.”* These three jointly accounted for 68.0% of all no-harm incidents, and resulted in interventions for the patients such as *“extra blood samples procedures, nursing care and treatments.” “Deficiencies in medication management”* were deemed to have a preventability rate twice as high as *“fall without harm”* and *“moderate pain.”* The most common potential contributing cause of *“fall without harm”* was *“deficiencies in nursing care, i.e., delayed, erroneous, omitted or incomplete care.”* For *“deficiencies in medication management”* and *“moderate pain”* the most common contributing cause was *delayed, erroneous, omitted or incomplete treatment*.

Today, patient safety researchers agree that the underlying causes of no-harm incidents and AEs are similar [23–25]. Therefore, detecting no-harm incidents can help us to establish preventable measures before an AE occurs. To our knowledge, no-harm incidents have not been investigated in home healthcare using RRR and TT methodology. We found only two studies from in-hospital settings using RRR for identification of no-harm incidents, [27, 28] which limits the possibilities for comparison, as does the fact that the care provided in a patient’s home environment is quite different from hospital care. *“Fall without harm”* was the most common no-harm incident, with less than half (40.9%) of the cases regarded as preventable. The critical difference in home healthcare compared with in-hospital care is that the patient and their relatives are largely autonomous, and the home healthcare environment is hard to control or standardize to the same extent as in-hospital settings. For example, it is difficult to remove stairs, thresholds or carpets without the consent of patients and relatives [6]. For that reason, prevention of falls (as well as prevention of other risks) in home healthcare is largely built upon a collaboration with patients and their relatives and a common understanding of risks. Studies demonstrate that patients and their relatives are a critical resource in identifying risks and preventing or mitigating incidents [32] that are rarely accounted for in medical records or incident reporting systems. This further indicates that the number of both AEs and no-harm incidents in home healthcare may be greatly underestimated. Schildmeijer et al. [28] found that the most common no-harm incidents in in-hospital care were related to drug therapy, with 87.9% of the cases regarded as preventable. This is comparable to our study, where *“deficiencies in medication management”* were regarded as preventable in as many as 98.4% of cases.

Within the three most common no-harm incidents, “*delayed, erroneous, omitted or incomplete treatment”* occurred as a contributing cause. Even in a controlled environment, like a surgical department, omission of a prescribed medication dose has been shown to be the second most common no-harm incident [27]. Although largely unexplored, there is reason to believe that deficiencies in medication management process is an important risk area or contributing cause of no-harm incidents, especially in home healthcare, where many different participants are involved in this process. In an earlier observational study of the medication management process in home healthcare, we found that deficiencies in the documentation systems made it easy to overlook or miss a prescribed dose, and such no-harm incidents were rarely documented in patient records [7]. Thus, measures to improve documentation, information transfer, as well as visualization of important information in patient records are warranted to identify and prevent no-harm incidents and AEs in relation to medication management. Further, in-depth analysis of omission problems may provide an alternative data source that could give some insight into blind spots that might be difficult to monitor in other ways. High-quality documentation is critical to RRR, as the result is dependent on sufficient qualitative documentation [22] to determine whether a no-harm incident has occurred and to assess its nature and preventability. In this study, no interventions were documented for a large proportion of the no-harm incidents, which may indicate that there is an underestimation of no-harm incidents.

The most commonly reported potential contributing cause of no-harm incidents in this study was “*delayed, erroneous, omitted or incomplete nursing care and treatment”*. Given that the combination of many minor flaws in the care of a patient is more often related to serious events [33] than any single dramatic failure, it is important to be aware of the underlying mechanisms for such deficiencies. These findings indicate a need to investigate how individuals and teams are supported and to improve working conditions, organizational conditions, and workplace culture [34]. Another potential contributing factor was *“lacking or deficient routines and guidelines or non-observance of existing routines/guidelines”*. In a complex system where the circumstances constantly change there is a risk that standardized guidelines do not work very well [35]. Safety is not a constant, but must be created by the professionals and other participants in the system. Resilient behavior in such an environment may be to switch between two disparate ways of working; on the one hand, to comply with standardized rules and guidelines, and on the other hand, to constantly adjust the activity to shifting situations that fall outside the scope of the rules and guidelines. In this study, some of the predefined contributing causes were broad. In order to gain deeper knowledge and to detect the systemic gaps concealed therein there is a need to further analyze how to categorize the potential contributing causes.

RRR has been successfully used to identify AEs in different contexts, recently also in home healthcare [20]. The method may be criticized for its reactive approach and linear thinking, which assumes that AEs are an effect of failures or have causes that can be found and fixed. However, the original Global Trigger Tool version [15] includes only events that have led to AEs related to acts of commission, focusing on safety as the absence of harmful events [36]. The main differences with our TT approach compared to the Global Trigger Tool are the adapted triggers for home healthcare, inclusion of both AEs [20] and no-harm incidents and acts of not only commission but also omission. The latter is recommended in a systematic review [13]. Harm and no-harm incidents can also result from poorly coordinated care or omission of care, sometimes over a long period of time. Many patients suffer harm, in the sense that their symptoms are untreated, through diagnostic errors and delays [37]. Additionally, events that cause only minor patient harm and no-harm incidents affect a patient’s psychological trust, recovery and participation, [38] and interventions to reduce such minor harm and no-harm incidents would probably have a positive impact on many patients. This study suggests that it is feasible to identify no-harm incidents and potential contributing causes such as omission of care using RRR with a TT, which means that an event may be identified and prevented before an AE arise. However, while a particular error, delay or omission may be the primary contributing cause of an incident, it is necessary to look at a wider context to detect the systemic gaps concealed therein [39, 40]. Such a broader approach to patient safety takes the complexity of today’s healthcare system into account, indicating that both good and bad outcomes emerge from interactions between people, organizations, technology, and the internal and external environment in a specific context [41]. Hibbert et al. [13] have recommended that omissions are included when using RRR.

### Strengths and limitations

The strengths of this study included having 600 records that were randomized from ten different sites, giving an overview of no-harm incidents occurring in home healthcare. To adapt to the patient perspective, we included all documented no-harm incidents regardless of caregiver, as this contributes to the identification of risk areas throughout the patient’s healthcare chain. Each review team included a small number professionals from the same workplace; it seems to strengthen inter-rater reliability that the reviewer group is small and its members work close together [12]. The reviewers analyzed records from their own organizations, which might bias the results. An advantage is local knowledge of the record system increasing the possibility to find events. On the other hand, there is a risk of underreporting due to inability to critically evaluate one’s own working place. A Swedish study found that reviewing you own institution resulted in more detected AEs [42]. This study also had more limitations to take into account. The possibility of detecting no-harm incidents and to trace the potential contributing causes is dependent on the quality of the documentation. The fragmented healthcare system with limited possibilities of getting access to a patient’s medical record across caregiver borders made it difficult to follow each patient’s care trajectory. The most common subcategories of potential contributing causes were too generic to identify concrete areas for improvement. Although, single interventions may not be enough to solve complex patient safety issues, many interventions in concert may have effect on multiple types of no-harm incidents. To increase the probability of selecting medical records with an AE or no-harm incident, we used a weighted sample and thus excluded patients with very limited home healthcare. This is in accordance with the GTT to only include admissions with at least 24 h length of stay [15]. Additionally, generalizability may be limited if home healthcare services have differing clinical standards.

## Conclusion

This study suggests that it is feasible to identify no-harm incidents and potential contributing causes such as omission of care using RRR with a TT adapted to the context. No-harm incidents and potential contributing causes are valuable sources of knowledge for improving patient safety, as they highlight system failures and indicate risks that may lead to AEs. The knowledge gained may be used to develop valid process indicators for systemic failures, as well as outcome indicators for structured evaluation and assessment of patient safety work in home healthcare. Such measures are highly warranted for proactive improvement and evaluation of healthcare services.

## Data Availability

The data are available from the corresponding author on reasonable request.

## Abbreviations

AE: Adverse event
NCC MERP: National Coordinating Council for Medication Error Reporting and Prevention
RRR: Retrospective record review
TT: Trigger tool

## References

[1] World Health Organization. Patient Safety: Making health care safer. Geneva: World Health Organization; 2017. https://apps.who.int/iris/handle/10665/255507. License: CC BY-NC-SA 3.0 IGO.

[2] Kohn LT, Corrigan JM, Donaldson MS. In: America. IoMCoQoHCi, ed. To Err is Human: Building a Safer Health System. Washington (DC): National Academies Press (US). 2000. 25077248

[3] Lamont T, Waring J (2015). Safety lessons: shifting paradigms and new directions for patient safety research. J Health Serv Res Policy.

[4] Lindblad M, Flink M, Ekstedt M (2018). Exploring patient safety in Swedish specialised home healthcare: an interview study with multidisciplinary teams and clinical managers. BMJ Open.

[5] Schildmeijer K, Wallerstedt B, Ekstedt M (2019). Healthcare Professionals’ Perceptions of Risk When Care Is Given in Patients’ Homes. Home Healthc Now.

[6] Lang A, Edwards N, Fleiszer A (2008). Safety in home care: a broadened perspective of patient safety. Int J Qual Health Care.

[7] Lindblad M, Flink M, Ekstedt M (2017). Safe medication management in specialized home healthcare - an observational study. BMC Health Serv Res.

[8] Beer JM, McBride SE, Mitzner TL (2014). Understanding challenges in the front lines of home health care: a human-systems approach. Appl Ergon.

[9] Naessens JM, Campbell CR, Huddleston JM (2009). A comparison of hospital adverse events identified by three widely used detection methods. Int J Qual Health Care.

[10] Christiaans-Dingelhoff I, Smits M, Zwaan L (2011). To what extent are adverse events found in patient records reported by patients and healthcare professionals via complaints, claims and incident reports?. BMC Health Serv Res.

[11] Classen David C, Resar R, Griffin F (2011). ‘Global trigger tool’shows that adverse events in hospitals may be ten Times greaterthan previously measured. Health Aff.

[12] Hanskamp-Sebregts M, Zegers M, Vincent C (2016). Measurement of patient safety: a systematic review of the reliability and validity of adverse event detection with record review. BMJ Open.

[13] Hibbert PD, Molloy CJ, Hooper TD (2016). The application of the global trigger tool: a systematic review. Int J Qual Health Care.

[14] Nilsson L, Borgstedt-Risberg M, Soop M (2018). Incidence of adverse events in Sweden during 2013–2016: a cohort study describing the implementation of a national trigger tool. BMJ Open.

[15] Griffin F, Resar R (2009). IHI Global Trigger Tool for Measuring Adverse Events. (Second Edition). IHI Innovation Series white paper.

[16] Blais R, Sears NA, Doran D (2013). Assessing adverse events among home care clients in three Canadian provinces using chart review. BMJ Qual Saf.

[17] Sears N, Baker GR, Barnsley J (2013). The incidence of adverse events among home care patients. Int J Qual Health Care.

[18] Johnson KG (2006). Adverse events among Winnipeg Home Care clients. Healthc Q.

[19] Lindblad M, Schildmeijer K, Nilsson L (2017). Development of a trigger tool to identify adverse events and no-harm incidents that affect patients admitted to home healthcare. BMJ Qual Saf.

[20] Schildmeijer KGI, Unbeck M, Ekstedt M (2018). Adverse events in patients in home healthcare: a retrospective record review using trigger tool methodology. BMJ Open.

[21] Genet N, Boerma WG, Kringos DS (2011). Home care in Europe: a systematic literature review. BMC Health Serv Res.

[22] Thomas EJ, Petersen LA (2003). Measuring errors and adverse events in health care. J Gen Intern Med.

[23] Reason J (2000). Human error: models and management. BMJ.

[24] Braithwaite J, Wears RL, Hollnagel E (2015). Resilient health care: turning patient safety on its head. Int J Qual Health Care.

[25] Hollnagel E (2011). Resilience engineering in practice : a guidebook.

[26] Wears RL, Hollnagel E, J B. The Resilience of Evereday Clinical Work. Dorchester United Kingdom Aschgate 2015.

[27] Paranagua TT, Bezerra AL, Silva AE (2013). Prevalence of no-harm incidents and edverse events in a surgical clinic. Acta Paul Enferm.

[28] Schildmeijer K, Unbeck M, Muren O (2013). Retrospective record review in proactive patient safety work - identification of no-harm incidents. BMC Health Serv Res.

[29] Swedish_Government. Health and Medical Services Act [Swe: Hälso- och Sjukvårdslag]. Stockholm: Socialdepartementet; 2017.

[30] World Health Organization (2009). Conceptual Framework for the International Classification for Patient Safety Version 1.1 Final Technical Report.

[31] National Coordinating Council for Medication Error Reporting and Prevention. NCC MERP Index for Categorizing Medication Errors. Available from: https://www.nccmerp.org/types-medication-errors. Retrieved 27 Mar 2020.

[32] Weingart SN, Pagovich O, Sands DZ (2005). What can hospitalized patients tell us about adverse events? Learning from patient-reported incidents. J Gen Intern Med.

[33] Vincent C, R A (2016). Safer Healthcare: Strategies for the Real World.

[34] Braithwaite J, Herkes J, Ludlow K (2017). Association between organisational and workplace cultures, and patient outcomes: systematic review. BMJ Open.

[35] Carthey J, Walker S, Deelchand V (2011). Breaking the rules: understanding non-compliance with policies and guidelines. BMJ.

[36] Runciman W, Hibbert P, Thomson R (2009). Towards an International Classification for Patient Safety: key concepts and terms. Int J Qual Health Care.

[37] Singh H, Meyer AN, Thomas EJ (2014). The frequency of diagnostic errors in outpatient care: estimations from three large observational studies involving US adult populations. BMJ Qual Saf.

[38] Brennan N, Barnes R, Calnan M (2013). Trust in the health-care provider-patient relationship: a systematic mapping review of the evidence base. Int J Qual Health Care.

[39] Wood D, Cook R (2002). Nine steps to move forward from error. Cogn Tech Work.

[40] Vincent C, Taylor-Adams S, Chapman EJ (2000). How to investigate and analyse clinical incidents: clinical risk unit and association of litigation and risk management protocol. BMJ.

[41] Holden LM (2005). Complex adaptive systems: concept analysis. J Adv Nurs.

[42] Unbeck M, Schildmeijer K, Henriksson P, Jürgensen U, Muren O, Nilsson L, Pukk HK (2013). Is detection of adverse events affected by record review methodology? an evaluation of the “Harvard Medical Practice Study” method and the “Global Trigger Tool”. Patient Saf Surg.

